# Developing and assessing a new web-based tapping test for measuring distal movement in Parkinson’s disease: a Distal Finger Tapping test

**DOI:** 10.1038/s41598-021-03563-7

**Published:** 2022-01-10

**Authors:** Noreen Akram, Haoxuan Li, Aaron Ben-Joseph, Caroline Budu, David A. Gallagher, Jonathan P. Bestwick, Anette Schrag, Alastair J. Noyce, Cristina Simonet

**Affiliations:** 1 Preventive Neurology Unit, Wolfson Institute of Population Health, Barts and The London School of Medicine and Dentistry, Queen Mary University of London, London, UK; 2 The Royal London Hospital, Barts Health NHS Trust, Whitechapel, London, UK; 3 Department of Clinical and Movement Neuroscience, UCL Institute of Neurology, London, UK

**Keywords:** Parkinson's disease, Diagnostic markers

## Abstract

Disability in Parkinson’s disease (PD) is measured by standardised scales including the MDS-UPDRS, which are subject to high inter and intra-rater variability and fail to capture subtle motor impairment. The BRadykinesia Akinesia INcoordination (BRAIN) test is a validated keyboard tapping test, evaluating proximal upper-limb motor impairment. Here, a new Distal Finger Tapping (DFT) test was developed to assess distal upper-limb function. Kinetic parameters of the test include kinesia score (KS20, key taps over 20 s), akinesia time (AT20, mean dwell-time on each key) and incoordination score (IS20, variance of travelling time between key taps). To develop and evaluate a new keyboard-tapping test for objective and remote distal motor function in PD patients. The DFT and BRAIN tests were assessed in 55 PD patients and 65 controls. Test scores were compared between groups and correlated with the MDS-UPDRS-III finger tapping sub-scores. Nine additional PD patients were recruited for monitoring motor fluctuations. All three parameters discriminated effectively between PD patients and controls, with KS20 performing best, yielding 79% sensitivity for 85% specificity; area under the receiver operating characteristic curve (AUC) = 0.90. A combination of DFT and BRAIN tests improved discrimination (AUC = 0.95). Among three parameters, KS20 showed a moderate correlation with the MDS-UPDRS finger-tapping sub-score (Pearson’s r = − 0.40, p = 0.002). Further, the DFT test detected subtle changes in motor fluctuation states which were not reflected clearly by the MDS-UPDRS-III finger tapping sub-scores. The DFT test is an online tool for assessing distal movements in PD, with future scope for longitudinal monitoring of motor complications.

## Introduction

Bradykinesia relates to the slowness of movement, and it is a core clinical sign of Parkinson’s disease (PD)^1^. Finger tapping tests have been widely utilised in neurophysiological examinations to assess upper extremity bradykinesia. Finger tapping is assessed visually on a 5-point rating scale, using the gold standard Movement Disorder Society Unified Parkinson’s Disease Rating Scale (MDS-UPDRS)^2^. Although the MDS-UPDRS-III is a comprehensive assessment, the integer scale prevents detection of subtle motor changes^3,4^ and inter-rater agreement is moderate at best^5^. Hence, a clear need for objective and consistent methods of assessing motor dysfunction exists.

Several methods focusing on objective finger tapping measurements have been explored. Hasan and colleagues described 33 of 47 technologies evaluating PD, focused on finger tapping movement; varying from optical systems, wearable sensors, electromagnetic motion capture devices and 3-D accelerometers and 3-D gyroscopes^6^. Unfortunately, little has translated to clinical practice either due to cost, practicality or lack of accuracy. More recently, the use of mobile smartphones and keyboard testing has gained traction, as a means of using digital biomarkers for remote and longitudinal monitoring. An up-to-date literature search was carried out to summarise recent quantitative measures of finger movement in PD from 2014 to 2021 (see Table 1). There is a growing interest in exploring the use of technology in PD. So far, a wide range of technology devices has been developed^7–29^. Smartphones are the most commonly used method (eight studies) followed by hand/tracker sensors (six studies), video recording (four studies), and 3D wearables (three studies); with only two tablet-based tests and one keyboard tapping test. Out of the 23 studies, 10 focused on alternative finger tapping movements, and the remainder focused on repetitive index finger tapping movement. All technologies focusing on index repetitive finger tapping required some form of specialised equipment (3D motion capture system, sensors or wearables). AUC and accuracy were not described in 13 studies, and correlation with motor scores of the UPDRS varied.

**Table 1:** Representative literature about quantitative measures of finger movements.

Reference	Test	Task	Sample	Parameters studied	Accuracy	Clinical correlation
Noyce et al. 2014^7^	BRAIN test: keyboard	ATT 30”	58 PD 93 AMC	KS^a^ AT IS	KS: 56% sensitivity, 80% specificity	KS—total UPDRS-III r = −0.53
Khan et al. 2014^8^	Computer vision framework and videos	FT	13 PD 6 HC		Accuracy 95%	NR
Kassavetis et al. 2015^9^	Smartphone application and accelerometer	ATT	14 PD	Tapping frequency Mean moving time Distance between taps	NR	Tapping frequency on the phone (r = − 0.75; P = 0.001), the mean moving time (r = 0.65; P = 0.001), and the distance between taps (r = − 0.61; P = 0.003
Maetzler et al. 2015^10^	Digitomotography	FT	33 PD 18 HC	IPI TF DEV	NR	IPI-UPDRS-III: r^2^ = 0.02, FT r^2^ = 0.01 TF-UPDRS-III: r^2^ = 0.02, FT r^2^ = 0.03 DEV-UPDRS: r^2^ = 0.16, FT r^2^= 0.16
Arora et al. 2015^11^	Smartphone	ATT	20 PD	Not specified: recorded voice, posture, gait, FT, reaction time test	Whole app PD vs controls mean sensitivity: 96.2% (SD 2%) mean specificity 96.9% (SD 1.9%) (finger tapping detail not given in isolation)	Mean error of whole app and UPDRS: 1.26
Sano et al. 2016^12^	PDFTsi—magnetic sensors	FT	21 PD	Distance, velocity, acceleration, interval	NR	Mean sequare error – 0.45
Lee et al. 2016^13^	Smartphone tapper	ATT (10”)	57 PD 87 HC	Number taps Amplitude^a^ Inter-tap distance Dwelling time	Total distance: AUC: 0.92 (95% CI 0.88–0.96) Dwelling time: AUC: 0.88 (95% CI 0.82–0.93)	Overall test—UPDRS-III r^2^ = 0.25 Overall test—UPDRS- FT sub-score r^2^ = 0.32
Ruzicka et al. 2016^14^	Contactless 3D motion capture system	FT (10”)	22 PD 22 HC	AvgFrq MaxOpV AmpDec	AmpDec: AUC = 0.87 MaxOpV: AUC = 0.81	MaxOpV-UPDRS-FT sub-score r = −0.48
Mitsi et al. 2017^15^	Tablet based application (iMotor)	ATT (30”)	19 PD 17 HC	Total taps, tap accuracy, velocity, interval, duration, reaction time	AUC 0.98 (0.93–1) 94% sensitivity, 93% specificity	UPDRS and tap accuracy: r = −0.35
Van den Noort et al. 2017^16^	Sensors—PowerGlove	FT	4 PD	FT, hand opening closing, pronation/supination	NR	NR
Gao et al. 2018^17^	PD-monitor (sensor)	FT (30”)	107 PD 49 HC 41 ET	EA-dynamical classifiers^b^	PD-monitor score: AUC = 0.89	Right side—MDS-UPDRS-FT: r = 0.82 Left side—MDS-UPDRS-FT: r = 0.78
Zhan et al. 2018^18^	Smartphone and ML	ATT	129 PD 0 HC	Voice, FT, gait, balance, reaction time	NR	Overall test—UPDRS r = 0.88, p < 0.001. Did not stratify different parameters
Lipsmeier et al. 2018^19^	Smartphone	ATT	44 PD 35 HC	Sustained phonation, rest tremor, postural tremor, finger-tapping, balance, and gait. Passive movements	PD vs controls p < 0.005. No AUC	NR
Prince et al. 2018^20^	Smartphone	ATT	312 PD 236 HC	Speed, rhythm, accuracy and fatigue	AUC 65.7%	NR
Butt et al. 2018^21^	Leap motion controller and different ML techniques	FT (10”)	16 PD 12 HC	Velocity, angle, amplitude, and frequency	Log regression 70.37% AUC 0.831. Naive Bayes 81.4% (AUC 0.811)	R: −0.72
Wissel et al. 2018^22^	Tablet	ATT	11 PD 11 HC	Total number of taps, tap interval, tap duration, and tap accuracy	ON vs OFF (0.60 ≤ AUC ≤ 0.82)	Tapping data and UPDRS effect moderate (−0.55 to 0.55)
Lee et al. 2019^23^	Leap motion controller (hand tracker)	FT	8 PD	Amplitude, frequency, velocity, slope and variance	NR	R = 0.86
Bobic et al. 2019^24^	Wearable sensors and 3D gyroscope	FT	13 PD 17 MSA 14 PSP 12 HC	Velocity, amplitude, amplitude decrement, hesitations and freezes, speed	NR	Test vs neurologists accuracy 82.69% + /- 2.72
Shin et al. 2020^25^	Conventional camera DL tracking algorithm	FT LA (10”)	29 PD 1 HC	Amplitude (mean, variability^a^) Interpeak interval (mean, variability^a^)	NR	FT – UPDRS-III: Interpeak interval var: r = 0.66 LA-UPDRS-III: Interpeak interval var: r = 0.7
Williams et al. 2020^26^	Smartphone camera DL tracking algorithm	FT (10”)	39 PD 30 HC	Speed Amp CV Rhythm	NR	r = 0.74 (speed in MBRS) r = 0.69 (three parameters combined)
Li et al. 2020^27^	3D FT measurement-sensor units and computer	FT	43 PD 30 HC	Motor coordination: slowness, amplitude, hesitation	NR	NR
Zhao et al. 2019^28^	Videos and time series clustering	FT	39 PD 30 HC	Decrement	NR	NR
Alberts et al. 2021^29^	Smartphone	ATT	23 PD	Number of taps, intertap interval and errors (double tapping)	NR	FT vs UPDRS: R = − 0.31, *p* = 0.04 Errors/freezing vs UPDRS: R = 0.44, *p* < 0.01; *R* = 0.43, *p* < 0.01, respectively

^a^Best parameter, *NR* not reported, *FT* finger tapping, *LA* leg agility, *ATT* alternating tapping test, between brackets: task duration in seconds, *PD* Parkinson’s disease, *HC* healthy controls, *AMC* age matched controls, *SWEDD* scan without evidence of dopamine deficiency, *ET* essential tremor, *MSA* Multi System Atrophy, *PSP* Progressive Supranuclear Palsy, *CV* coefficient variance, *KS* kinesia score, *AT* alternating score, *IS* incoordination score, *IPI* Interpeak Interval, *TF* Tap Force, *DEV* Tap Deviation, ^b^*EA* evolutionary algorithms (^b^a form of artificial intelligence ^b^using an objective score scaled from – 1 to + 1 where higher scores indicate greater severity of bradykinesia), *MOV* maximum opening velocity, *TD* total distance, ^b^average frequency (AvgFrq), maximum opening velocity (MaxOpV) and amplitude decrement (AmpDec), *PDFTsi* Parkinson’s disease finger-tapping severity index using magnetic sensors, *DL* Deep Learning, *ML* Machine Learning,* MBRS* Modified Bradykinesia Rating Scale. r^2^: coefficient of determination for simple regression analysis, r: Pearson correlation coefficient, R: Spearman's rank correlation.

The BRadykinesia Akinesia INcoordination (BRAIN) test is a previously validated online keyboard tapping test for detecting upper-limb motor dysfunction^7,30^. The test requires participants to alternately tap the ‘S’ and ‘;’ keys on a computer keyboard using one index finger, as fast and accurately as possible, for 30 s^7^. As movement arises at the level of the elbow and shoulder, the test captures proximal movement. Existing literature suggests that proximal and distal movements are governed by two distinct neural pathways^31,32^. This possibly explains why, as a diagnostic test, the BRAIN test historically demonstrates a relatively low detection rate (sensitivity) for PD (58–65%) given high specificity (81–88%)^33^. Additionally, the BRAIN test requires significant hand–eye coordination, which may be unsuitable for patients with visual impairment^33^.

Thus, to address these gaps, a new Distal Finger Tapping (DFT) test was developed; to objectively and remotely assess distal index repetitive finger tapping movement, without the requirement for specialised equipment. The aims of this study are to demonstrate proof-of-concept and investigate whether the DFT test correlated with MDS-UPDRS finger tapping subscores, differentiates PD patients from controls and whether it has potential in monitoring motor fluctuations.

## Methods

### Distal Finger Tapping (DFT) test

The DFT test is an online tapping test, compatible with regular laptops and computers with a keyboard, accessed by participants using unique tokens (via https://predictpd.com/en/braintest).

**Figure 1:**
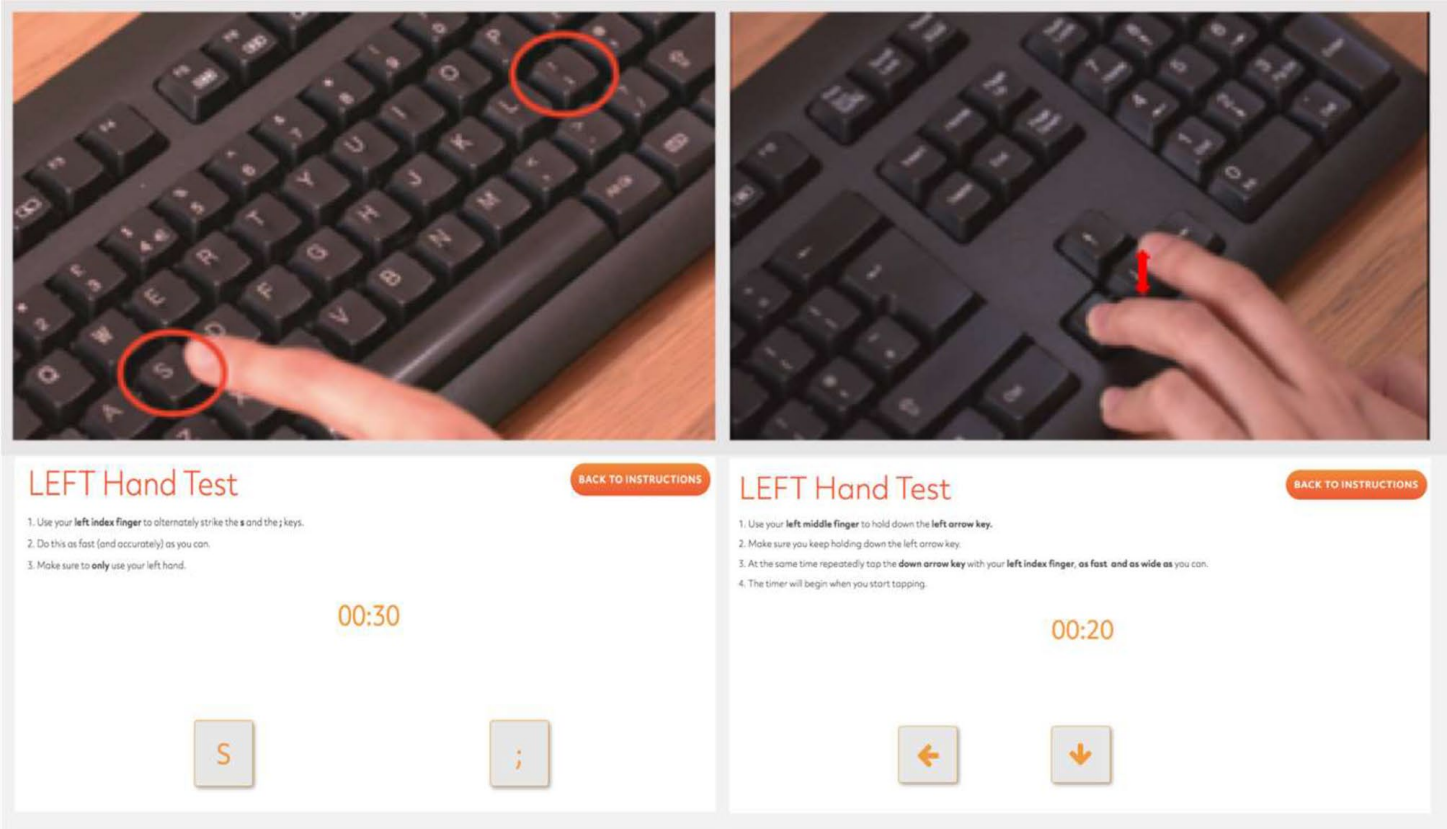
Comparison of the BRAIN test and DFT test. Left: BRAIN test, alternate tapping of the ‘s’ and ‘;’ keys with the index finger and online interface below. Right: DFT test, repeated tapping of down arrow key with left index finger whilst depressing the left arrow key with left middle finger and online interface below.

Participants were instructed to repeatedly tap the down arrow key with their left index finger, as fast as possible for 20 s, whilst simultaneously depressing the left arrow key with their left middle finger. The same task was then repeated for the right hand. These instructions stabilise the wrist and forearm, isolating movement to the index finger metacarpal joint, thereby giving a true measurement of distal finger movement (see Fig. 1). This movement is also similar to the MDS-UPDRS finger tapping task where patients are asked to tap their index finger and thumb repeatedly.

Three kinetic parameters were generated by the DFT test: kinesia score (KS20), the number of keystrokes in 20 s reflecting speed; akinesia time (AT20), average dwell time that keys are depressed reflecting akinesia; and incoordination score (IS20), the variance of travelling time between keystrokes reflecting rhythm.

### Participants

This study had two distinct parts. In the first stage of the study, patients fulfilling the Queen Square Brain Bank criteria for PD were consecutively recruited from the Movement Disorder clinic at the Royal London Hospital (RLH) between February and August 2019. Healthy controls were recruited (either relatives of patients or staff from RLH), and a subset of controls was sourced from the PREDICT-PD study. For the second stage, PD patients taking dopaminergic treatment and usually experiencing motor fluctuations were recruited from the same clinic (fluctuation states were based on clinical impressions, rather than levodopa timing). Exclusion criteria included any comorbidities that could interfere with the performance of the task, such as arthritis, previous stroke, dementia, and severe dyskinesias. Controls were excluded if they had any known neurological disorder.

Participants were seated in front of a computer/laptop, where they were asked to read on-screen instructions and independently complete the DFT and BRAIN test, only the first successful test was recorded for each patient. Total MDS-UPDRS-III scores were recorded for PD patients by trained individuals (NA & ABJ). The patients’ subjective clinical state was recorded, with ‘On’ defined as a functional state when there is a good response to medication, and ‘Off’ defined as a poor functional state despite taking medication or after the symptomatic effect of medication had passed. To avoid patients coming to the hospital another day, recruited patients participated in the study on the same day, hence, it was not logistically possible to control time between levodopa administration and testing. Additionally, to investigate the presence of a learning effect, seven of the healthy controls completed the DFT test five times within a 3-h period.

The second part of the study evaluated the use of the DFT test in assessing motor fluctuations. In order to capture real-life motor fluctuations, assessments were carried out in patients’ homes. Patients performed the DFT and BRAIN test, alongside MDS-UPDRS-III assessment carried out by the same trained neurologist (CS) under the same conditions. Assessments were organised to coincide with the timings of patients’ usual motor fluctuations and clinical impressions during home visits further confirmed patients’ fluctuation states. Adjustments were made to the schedule where necessary (i.e. waiting for patients’ medication effect to wear off in order to test the ‘Off’ state). Four patients were invited to complete the DFT test asynchronously on further occasions at home, for longitudinal monitoring of motor fluctuations. As it was not possible for in-person corroboration of functional states, patients were instructed to complete the test according to their subjective impressions—patients recognised their ‘On’ state as when levodopa was effective and motor symptoms were controlled, and ‘Off’ state as when levodopa was ineffective and motor symptoms re-emerged.

### Statistical analysis

Normality was assessed using D'Agostino and Pearson test/ Shapiro–Wilk test. Descriptive statistics were calculated for all three parameters (KS20, AT20, IS20), with mean and standard deviation (SD) being reported for normally distributed data and median and interquartile range (IQR) for not normally distributed data. DFT test scores in patients and controls from the first stage of study were compared using the unpaired t-test and Mann–Whitney U test for parametric and non-parametric data respectively. Receiver operating characteristic (ROC) curves were generated using Wilson/Brown method, determining sensitivity and specificity of parameters. Logistic regression and ROC curves defined AUC values for combination analysis of DFT and BRAIN test variables. The relationship between test parameters and MDS-UPDRS-III were assessed using Pearson’s correlation and Spearman’s rank correlation. In controls, one-way repeated measures ANOVA was used to detect a learning effect. Intra-Class Correlation coefficient (ICC) for test–retest reliability was calculated using consistency, two-way mixed effects. Interpretation of ICC values was based on recommendations by Koo and Li^34^. Paired t-tests and Wilcoxon matched-pairs signed rank tests were conducted using the best recorded ‘On’/‘Off’ state to investigate whether the DFT test and MDS-UPDRS-III could differentiate between fluctuations. Further, mixed effect models were used to define the effects of ‘On’ and ‘Off’ state on each outcome measure (MDS-UPDRS-III finger tapping sub-score, KS20, AT20, IS20, KS30, AT30 and IS30). Each model included therapy state (2-levels: ‘On’ and ‘Off’ state) as a fixed effect and subject number and trial number as random effects. The significance level for all calculations was set as p < 0.0025 (derived by Bonferroni calculation to reduce type 1 error). All data were analysed using GraphPad Prism version 8.0.2, IBM SPSS version 27 and Stata version 15.

Informed consent was obtained from all participants and/or their legal representatives. All methods were performed in accordance with the relevant guidelines and regulations.

### Ethics approval

Ethics approval was granted by the Queen Square Research Ethics Committee (09/H0716/48).

### Consent to participate and for publication

Informed consent was obtained from all participants and/or their legal representatives.

## Results

Fifty-five PD patients and sixty-five frequency-matched controls were included in the first stage of the study. Individuals with PD and controls had similar age and gender (p > 0.05). For patients taking levodopa, 48 considered themselves to be ‘On’ and 2 ‘Off’ whilst completing the test, on average patients took levodopa 150 min before completing the test. Table 2 summarises the demographic data for participants from the first stage of the study.

**Table 2:** Baseline characteristics of PD patients and controls from the first stage of the study.

	PD	Controls
Number	55	65
Mean age (SD)	66.8 (9.6)	71.2 (9.5)
**Gender**		
Female	24 (44%)	36 (55%)
Male	31 (56%)	29 (45%)
**Education**		
Primary	3 (5%)	–
Secondary	24 (44%)	–
Higher	10 (18%)	–
Further	18 (33%)	–
**Occupation**		
Professional	25 (46%)	–
Non-professional skilled	9 (16%)	–
Non-professional/non-skilled	21 (38%)	–
Mean yrs since PD diagnosis (SD)	6.3 (4.9)	–
**Most affected side**		
Right	17 (31%)	–
Left	36 (65%)	–
Equally	2 (3%)	
**Levodopa**		
Yes	50 (91%)	–
No	5 (9%)	–
Median minutes since levodopa dose (IQR)	150 (60, 210)	–
% Patients who had taken Levodopa < 12 h	50 (100%)	
**On/Off**^a^		
On	48 (96%)	–
Off	2 (4%)	–
**Mean MDS-UPDRS-III total (SD)**		
On (n = 48)	38.2 (16.4)	
Off (n = 2)	67.5 (6.4)	
**Mean MDS-UPDRS finger tapping subscore (SD)**		
Off (n = 48)	3.8 (1.6)	
On (n = 2)	5.5 (0.7)	

Also see summary characteristics for PD patients of the second stage of the study.

^a^On/Off refers to the question in the MDS-UPDRS, which asks whether patients taking levodopa could notice the effect of medication at the time of examination, 5 patients were not taking any levodopa.

In the second stage, nine additional PD patients were recruited for monitoring motor complications (mean age in years ± SD: 62.78 ± 7.10, mean disease duration in years ± SD: 9.00 ± 5.52 and gender distribution: 5 male and 4 female patients). One patient was excluded from daytime monitoring with MDS-UPDRS-III analysis due to an unexpected lack of fluctuations on the day of assessment. During the home visits, all patients had taken levodopa ≤ 12 h (4.03 h since their last levodopa dose on average) and achieved an ‘On’ state of 1.17 h on average after taking levodopa. Four patients with fluctuations agreed to carry out independent remote testing to monitor their daytime motor fluctuations.

Associations between DFT test parameters with age and gender were assessed in control subjects (see Table S1 in Supplementary material). Neither age nor gender overtly affected the test parameters. The performance between dominant and non-dominant hand was different in the control group, thus the average of both hands was used for analysis. Since PD has an asymmetric pattern, the most affected side in PD cases was selected. The identification of the most affected side was based on the side with the worst MDS-UPDRS-III scores.

### Comparison between PD patients and controls

**Figure 2:**
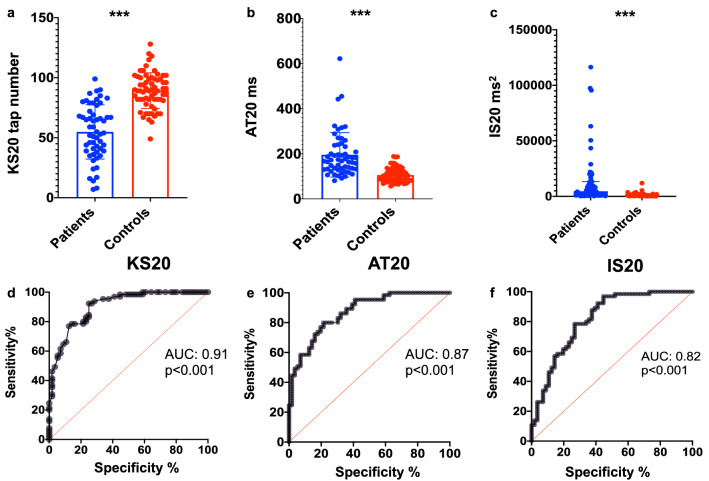
Comparison of KS20, AT20 and IS20 in PD patients and controls. Spread of (**a**) KS20, (**b**) AT20 (mean and SD) and (**c**) IS20 (median and IQR) for patients and controls. Receiver operating curves for (**d**) KS20, (**e**) AT20 and (**f**) IS20.

**Figure 3:**
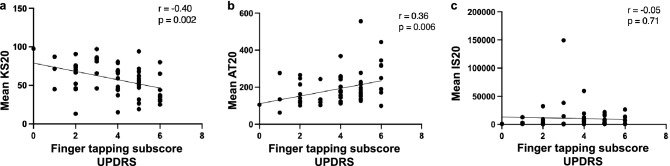
Correlation between DFT parameters and MDS-UPDRS-III finger tapping sub-socres. (**a**) Moderate negative correlation with KS20 and UPDRS. (**b**) Moderate positive correlation seen with AT20 and finger tapping sub-score. (**c**) No correlation seen with IS20 and finger tapping sub-score.

All three DFT parameters discriminated between patients (n = 55) and controls (n = 65). KS20 was the best discriminator, with 79% sensitivity for 85% specificity and an AUC of 0.90 (95% CI 0.85–0.96). The corresponding sensitivities for 85% specificity for AT20 and IS20 were 68% and 57%, with respective AUC’s of 0.87 (95% CI 0.81–0.93) and 0.82 (95% CI 0.74–0.89) (see Table 3 and Fig. 2). BRAIN test KS20, AT20, IS20 achieved AUC of 0.89, 0.88, and 0.71 respectively. The combination of DFT parameters improved discrimination with an AUC of 0.92 (80% sensitivity with 84% specificity, at 0.5 probability cut-off), and the combination of both DFT and BRAIN test parameters gave an AUC of 0.95 (80% sensitivity and 94.5% specificity, at 0.6 probability cut-off) (see Table S2 in Supplementary material). A moderate correlation was found for KS20 and AT20 against MDS-UPDRS-III finger tapping sub-scores (Pearson’s r = − 0.40, p = 0.002, and r = 0.36 p = 0.006) (see Fig. 3).

**Table 3:** Comparison of DFT KS20, AT20, and IS20 between patients and controls and corresponding ROC analysis.

	Mean KS20 (95% CI)	Mean AT20 (95% CI)	Median IS20 (IQR)
PD (n = 55)	55.0 (48.9, 61.1)	195.6 (168.7, 222.5)	4589 (1137, 13,464)
Controls (n = 65)	89.3 (85.6, 93.0)	105.5 (97.5, 113.4)	779.5 (357.7, 779.5)
p value	< 0.001^a^	< 0.001^a^	< 0.001^b^

	KS20 Sensitivity	AT20 Sensitivity	IS20 Sensitivity
Specificity—90% (cut-off)	66.2% (82.5)	58.5% (108.2)	46.2% (717.6)
Specificity—85% (cut-off)	78.5% (80.5)	67.7% (116.4)	56.9% (853.4)
Specificity—80% (cut-off)	78.5% (78.5)	76.9% (127.9)	61.5% (957.3)
Area under curve the ROC curve (95% CI)	0.90 (0.85, 0.96)	0.87 (0.81, 0.93)	0.82 (0.74, 0.89)

KS20, kinesia score; AT20, akinesia time; IS20, incoordination score; CI, confidence interval; IQR, interquartile range; SD, standard deviation.

^a^Unpaired t-test.

^b^Mann–Whitney test. Plotted in Fig. 2.

Repeat testing in seven of the controls did not reveal any learning effect in KS20 (p = 0.53), AT20 (p = 0.58) or IS20 (p = 0.24) using one-way repeated measures ANOVA. ICC values for test–retest reliability showed excellent reliability for all 3 parameters (KS20 ICC = 0.91, AT20 ICC = 0.92, IS20 ICC = 0.93).

### Monitoring motor fluctuations

**Figure 4:**
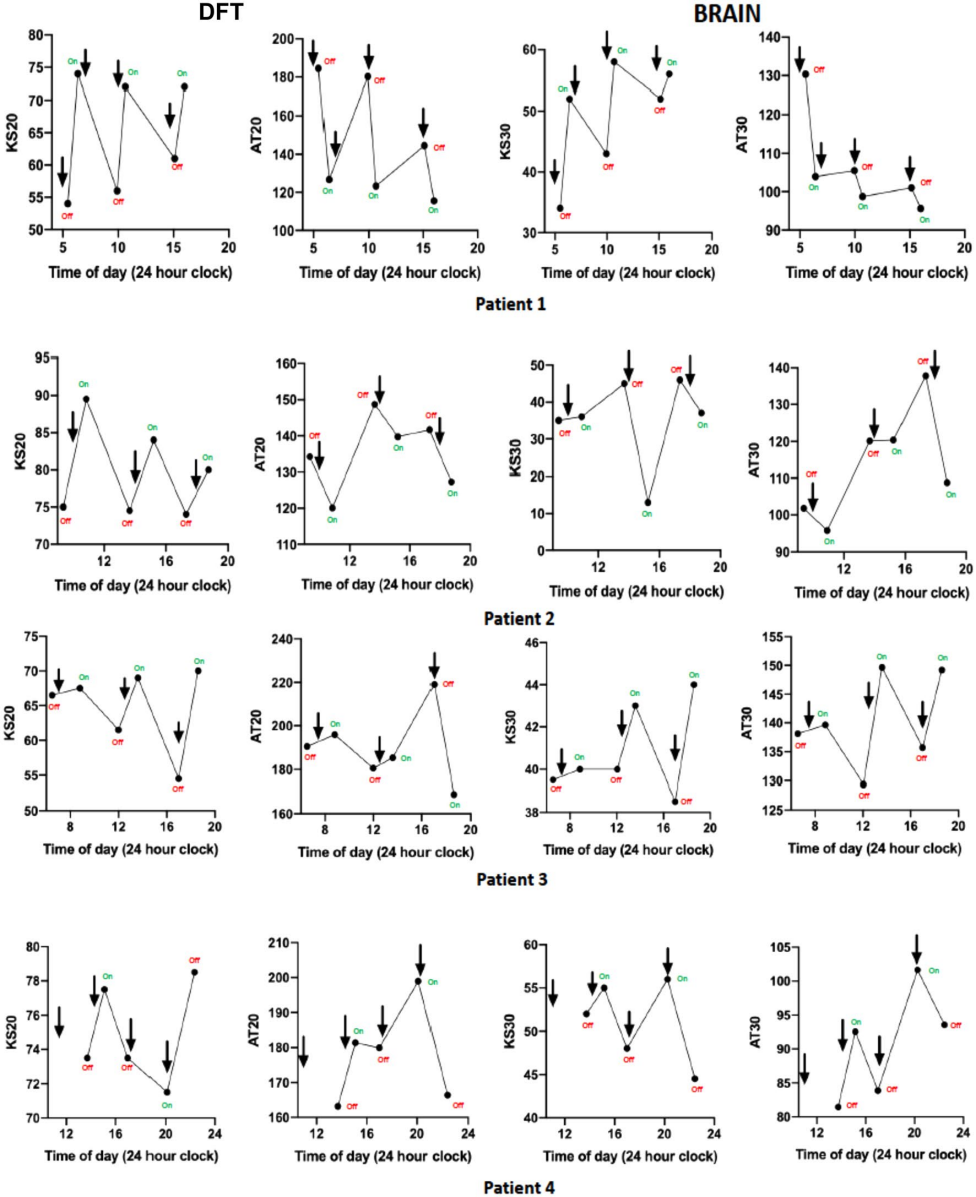
Repeat testing in 4 PD patients with predictable motor fluctuations using the DFT and BRAIN test. Dots represent when the test was completed, and arrows denote the time when levodopa was taken. KS20 (DFT test) and KS30 (BRAIN test) scores are expected to increase in the ‘On’ state, whereas AT20 and AT30 scores are expected to decrease in the ‘On’ state.

The DFT test provided suggestive evidence for a difference between patients’ subjective ‘On’ and ‘Off’ states using KS20 and IS20, although neither difference was statistically significant (KS20 ‘Off’—62.78 (95% CI 50.06–75.50) vs KS20 ‘On’—71.78 (95% CI (61.49–82.07), p = 0.05; IS20 ‘Off’—3452 (95% CI (1833–20,178) vs IS20 ‘On’—1232 (95% CI (845.3–10,017), p = 0.04; see Table 4). Contrastingly, the finger tapping sub-score of the MDS-UPDRS-III showed no significant differences between their ‘On’ and ‘Off’ states (mean FT sub-score ‘Off’: 2 (95% CI 1.37–2.63) vs mean FT sub-score ‘On’: 1.5 (95% CI 0.73–2.27), p = 0.10; see Table 4). Further, mixed effect models were used to measure the effects of therapy (2-level fixed effect: ‘On’ and ‘Off’ state) on each motor outcome (test parameters and finger tapping sub-score). The effect of therapy was defined based on the variability of parameters across all trials (random effects). KS20 was found to have the strongest correlation with ‘On’ and ‘Off’ states being almost 7 times higher in ‘On’ state compared with ‘Off’ state (coefficient = 6.7, 95% CI 2.42–11.09; see Table S3 in Supplementary material). This result was in agreement with what is represented in Fig. 4: amongst the four patients who completed the tapping tests more than twice, KS20 was found to be the most consistent parameter with subjective motor fluctuations (see Fig. 4). In **patient 1**, KS and AT scores from the DFT and BRAIN test fluctuated during the day and were in agreement with subjective ‘On–Off’ motor states. In **patient 2**, KS20 scores performed during ‘On’ states progressively decreased throughout the day, whilst remaining relatively constant across ‘Off’ periods. However, this pattern was not reflected in BRAIN test parameters. Of note, in **patient 4**, the KS20 score did not improve following the third levodopa dose, possibly reflecting an additional ‘No On’ or ‘Delayed On’, which again was not detected by the BRAIN test.

**Table 4:** Comparison of KS20, AT20 and IS20, UPDRS finger tapping (FT) subscores between patients’ ‘On’ and ‘Off’ states.

Parameter	PD ‘Off’	PD ‘On’	p-value
Mean KS20 in taps (95% CI)	62.78 (50.06, 75.50)	71.78 (61.49, 82.07)	0.05^a^
Mean AT20 in msec (95% CI)	155.0 (118.3, 191.7)	153.1 (120.6, 185.7)	0.88^a^
Median IS20 in msec^2^ (IQR)	3452 (1833, 20,178)	1232 (845.3, 10,017)	0.04^b^
Mean MDS-UPDRS-FT (95% CI)	2 (1.37, 2.63)	1.5 (0.73, 2.27)	0.10^a^

KS20, kinesia score; AT20, akinesia time; IS20, incoordination score; CI, confidence interval; IQR, interquartile range.

^a^Two-tailed paired t-test.

^b^Wilcoxon matched-pairs signed rank test.

## Discussion

In this proof-of-concept study, we demonstrated that a simple keyboard finger tapping test can be used to accurately monitor distal finger movements, distinctive from proximal action. The DFT test is able to quantify separate components of distal movement such as speed, akinesia, and rhythm. Interestingly, all three DFT test parameters alone were able to significantly distinguish patients from controls, with the combination of all three DFT test parameters demonstrating an accurate diagnostic performance. Moreover, both KS20 and AT20 from the DFT test significantly correlated with finger-tapping sub-score.

In contrast to MDS-UPDRS finger tapping sub-scores, the DFT test showed promise in detecting motor fluctuations. The DFT demonstrated a better correlation with subjective motor fluctuations than the BRAIN test, suggesting that distal motor impairment might have a stronger impact on patients’ quality of life. This also reinforces the idea that distal and proximal movements may be differentially affected in PD^35–38^. Whilst these findings are exploratory, both tapping tests show potential in objectively capturing daily symptom oscillations. They may provide clinicians with a clearer understanding of patients’ subjective interpretations of fluctuation states, enabling individualised tailoring of management plans. These findings warrant further analysis, it may be worth examining how well patients’ subjective impressions correlate with these objective measures.

Although objective tests have been developed previously, few have been designed to discriminate between distal and proximal movements. Distal and proximal bradykinesia have been demonstrated to be differentially affected in PD and have distinct responses to therapeutic options^39^. Used in conjunction with the BRAIN test, the DFT test would provide a complementary view of proximal and distal upper-limb movement. In contrast to the BRAIN test, the DFT test eliminates complex hand–eye coordination. Moreover, as subjects were instructed to repeatedly tap the same key, this prevented a ‘paradoxical kinesia’ phenomenon^40^ which could be present in the BRAIN test whereby the process of actively finding two separate keys potentially acts as a visual clue.

Unlike previous tests, the DFT test is devoid of specialist equipment or software and is freely available online, hence can be accessed by any laptop or computer. Further, the test only requires less than 1 min in total, thereby increasing compliance and versatility. Patients of a differing educational background, were able to independently complete the test, increasing applicability. Results are automatically transferred to a secure and central server for clinicians to view, and data can only be accessed using unique tokens and passwords, adding to safety of patient data. This is in contrast to previous tapping tests where cost, feasibility and compliance have limited clinical translation^6^. Practically, the DFT test serves as a key strength for unsupervised remote assessment of patients’ longitudinal motor function, which would prove particularly useful in the COVID-19 era where face-to-face clinic appointments are substantially reduced.

Despite the potential applicability of the DFT test, it entails several limitations. Importantly, the DFT test is not able to measure amplitude and its decrement. Which further limits the IS as a reliable parameter, as the coefficient of variation of travelling time between keystrokes is unable to be derived. Practically, it is not feasible to capture amplitude with a keyboard alone and using the DFT test as a remote assessment tool would not favour the incorporation of additional equipment. For that reason, it cannot be said that the DFT test comprehensively measures bradykinesia, as amplitude is an essential component of its definition. Nevertheless, the DFT test was able to accurately capture relevant kinetic aspects of distal movement such as frequency, rhythm and velocity. It is important to note that the DFT test cannot be considered a holistic measure of motor function, as it cannot account for other components of movement known to be affected in PD, such as walking, speech, and facial expression; thus its aim is not to act as a diagnostic tool but rather to complement clinical expertise. Moreover, whilst two parameters significantly correlated finger-tapping subscores were significant, the findings were only moderate. Additionally, IS20 did not correlate with finger tapping subscores. This is possibly due to the crude integer scale UPDRS, which is not able to capture subtle effects such as the variance of travelling time. Hasan et al. described that correlation of MDS-UPDRS is not a good indicator for objective monitoring of tests. Instead, clinimetric properties such as test–retest reliability, sensitivity, specificity, responsiveness, feasibility and administrative burden should be considered to carry out a more comprehensive evaluation of these tests, all of which the DFT test bodes well in.

In the analysis of the DFT test in motor fluctuations, a 12-h washout of levodopa was not implemented due to ethical considerations of disabling ‘Off’ state complications. Existing literature also notes that patients can experience prolonged motor improvement following levodopa, due to ‘long-duration response to levodopa (LDR)’, thus rendering overnight withdrawal unreliable^41^. A further limitation faced by the DFT as a longitudinal monitoring tool is the potential for confounding factors such as mood and alertness to influence patients’ subjective interpretations of ‘On’ and ‘Off’ states, as opposed to it being based solely on motor function. Although it was not possible to control these external factors, the results aimed to represent the ‘real-life’ situation of remote monitoring in patients. Similarly, the use of different keyboard types with varying key sizes and resistance was not considered in the present study and may have interfered with the performance of the task.

Finally, selecting the most affected side in PD could have magnified the accuracy of the test. However, this was carried out as PD is an asymmetrical condition and thus it would be appropriate to assess the performance of the tapping test in patients’ worst affected side.

Future directions for the DFT test include assessing it in combination with the BRAIN test as a form of remote longitudinal monitoring of patients’ upper-limb function, which may help to facilitate treatment adjustments. Additionally, the DFT test serves valuable research purposes, by contributing to a wider set of motor batteries than can be used collectively for follow-up of asymptomatic individuals who are at high and low risk of developing PD. It is currently incorporated into PREDICT-PD, a population-based longitudinal study with the aim of detecting early motor manifestations^42^.

## Conclusion

The DFT test offers a remote and objective method of capturing distal upper-limb function. Further work is warranted to validate the DFT test as a supplementary clinical tool for diagnosis and remote monitoring of PD motor complications.

## Supplementary Information

Supplementary Tables.

## Data Availability

Data and materials are readily available, if required.
