# Supplementary material for: Developing and assessing a new web-based tapping test for measuring distal movement in Parkinson’s disease: a Distal Finger Tapping test
Source: Sci Rep. 2022 Jan 10;12:386. doi: 10.1038/s41598-021-03563-7 (PMC8748736; doi:10.1038/s41598-021-03563-7)
Supplement: Supplementary file 1 — Supplementary Tables. [file 41598_2021_3563_MOESM1_ESM.docx]

**Supplementary material**

**Table S1. Analysis of characteristics that influence KS20, AT20 and IS20 in controls**

|  |  | Mean KS20* | **p-Value** | Mean AT20* | **p-value** | Median IS20* | **p-value** |
| --- | --- | --- | --- | --- | --- | --- | --- |
| **Mean Age** | 71.2 | r=-0.05 | 0.67^a^ | r=-0.03 | 0.80^a^ | r=0.10 | 0.42^b^ |
| **Gender** |  |  |  |  |  |  |  |
| **- Female** | 36 | 84.2 | 0.49^c^ | 121.4 | 0.06^c^ | 1217 | 0.52^d^ |
| **- Male** | 29 | 86.6 |  | 104.5 |  | 1186 |  |

KS20, kinesia score; AT20, akinesia time; IS20, incoordination score *Mean and medians given except for associations with age where correlation coefficient (r) is given. ^a^Pearson; ^b^Spearman; ^c^Unpaired t-test; ^d^Mann-Whitney, ^e^Paired t-test, ^f^Wilcoxon test

**Table S2. Sensitivity and specificity for combination analysis of DFT and BRAIN test parameters.**

|  | **DFT test parameter combination** | | **DFT and BRAIN test combination** | |
| --- | --- | --- | --- | --- |
| **Probability cut-off** | **Sensitivity (%)** | **Specificity (%)** | **Sensitivity (%)** | **Specificity (%)** |
| 0.4 | 81.8 | 81.8 | 89.1 | 85.5 |
| 0.5 | 80.0 | 83.6 | 85.5 | 87.3 |
| 0.6 | 70.9 | 90.9 | 80.0 | 94.5 |
| 0.7 | 61.8 | 92.7 | 76.4 | 98.2 |
| 0.8 | 58.2 | 98.2 | 65.5 | 98.2 |
| 0.9 | 52.7 | 98.2 | 60.0 | 98.2 |

**Table S3. Mixed effect models examining the effect of fluctuation states (‘On’ and ‘Off’) on outcome measures.**

| **Outcome measure** | **Coefficient** | **95% CI** |
| --- | --- | --- |
| KS20 | 6.76 | 2.42 – 11.09 |
| KS30 | 2.28 | -2.19 – 6.76 |
| AT20 | -7.54 | -20.24 – 5.16 |
| AT30 | -5.78 | -16.92 – 5.36 |
| IT20 | -3376.83 | -7267.09 – 513.45 |
| IT30 | 5801.40 | -7809.68 19412.48 |
| MDS-UPDRS-FT | -0.76 | -1.07 – -0.46 |
| KS20/30, kinesia score; AT20/30, akinesia time; IS20/30, incoordination score; CI, confidence interval. Mixed effect model coefficient. Fixed effect: ‘On’ and ‘Off’ state. Random effect: number of trials. | | |
